# CO and NO_2_ Selective Monitoring by ZnO-Based Sensors

**DOI:** 10.3390/nano3030357

**Published:** 2013-07-05

**Authors:** Mokhtar Hjiri, Lassaad El Mir, Salvatore Gianluca Leonardi, Nicola Donato, Giovanni Neri

**Affiliations:** 1 Laboratory of Physics of Materials and Nanomaterials Applied at the Environment, Faculty of Sciences, University of Gabes, Gabes 6072, Tunisia; E-Mails: m.hjiri@yahoo.fr (M.H.); lassaad.elmir@fsg.rnu.tn (L.E.); 2 Department of Physics, College of Sciences, Al Imam Mohammad Ibn Saud Islamic University (IMSIU), Riyadh 11623, Saudi Arabia; 3 Department of Electronic Engineering, Chemistry and Industrial Engineering, University of Messina, Messina 98166, Italy; E-Mails: leonardis@unime.it (S.G.L.); ndonato@unime.it (N.D.)

**Keywords:** ZnO, nanoparticles, nanofibers, gas sensor, CO, NO_2_, sol-gel, electrospinning

## Abstract

ZnO nanomaterials with different shapes were synthesized, characterized and tested in the selective monitoring of low concentration of CO and NO_2_ in air. ZnO nanoparticles (NPs) and nanofibers (NFs) were synthesized by a modified sol-gel method in supercritical conditions and electrospinning technique, respectively. CO and NO_2_ sensing tests have demonstrated that the annealing temperature and shape of zinc oxide nanomaterials are the key factors in modulating the electrical and sensing properties. Specifically, ZnO NPs annealed at high temperature (700 °C) have been found sensitive to CO, while they displayed negligible response to NO_2_. The opposite behavior has been registered for the one-dimensional ZnO NFs annealed at medium temperature (400 °C). Due to their adaptable sensitivity/selectivity characteristics, the developed sensors show promising applications in dual air quality control systems for closed ambient such as automotive cabin, parking garage and tunnels.

## 1. Introduction

In recent years, research activity in the field of gas sensing has been greatly amplified. This interest is mainly due to problems related to pollution and strict regulations for safety taken by several countries in many industrial sectors. Metal-oxide semiconductor gas sensors have attracted great attention due to their advantageous features, such as high sensitivity under ambient conditions, low power consumption and simplicity in fabrication. Although metal oxides have been demonstrated to be sensitive to many gases, e.g., O_2_, H_2_O, ethanol, methanol, CO, NO_2_, NH_3_, *etc.*, one of the great challenges of using these materials is the selectivity, *i.e.*, how they can differentiate the gas of interest from other gases [1,2].

Among them, ZnO has been shown to be useful material for monitoring various pollutant gases like CO, benzene, NO*_x_*. Zinc oxide is an n-type direct band semiconductor with a wide band gap (3.3 eV) and find wide ranging applications in varistors [3], surface acoustic wave (SAW) devices [4], transparent conducting oxide electrodes [5], solar cells [6], blue/UV light emitting devices [7], gas sensors [8,9], *etc.* Its conductivity can be tailored by controlling the deviation from stoichiometry and by doping [10]. For gas sensing applications, ZnO in pure and doped form has been intensively studied [9,11].

Recent reports show that changes in particle morphology (shape) can dramatically alter electrical characteristics and reactivity of ZnO when interacting with gaseous species [12]. The links between morphology, surface termination, and electrical response are complex and not yet well understood at a predictive level. Thus, experimental studies that build connections between particle morphology, surface reactivity and bulk electrical properties can provide crucial insights to develop robust strategies to improve the sensitivity and selectivity of metal-oxide gas sensors.

In this study, ZnO nanomaterials with different shapes were synthesized, characterized and tested in the monitoring of low concentration of NO_2_ and CO in air. The monitoring of CO and NO_2_ is of utmost importance in the environmental control. CO is a gas produced during incomplete combustion and it is toxic at very low concentrations. NO_2_ is another environmental pollutant arising from combustion facilities. In confined environments, such as automotive cabin, garage parking or tunnels, high concentrations of these pollutants can create serious hazard for the health and should be continuously monitored and controlled [13]. The concentrations of pollutants to be monitored in these ambient are very low, 5–50 ppm for CO whereas levels of NO_2_ are in the sub-ppm range [14].

Our final aim is to develop sensors to be used in devices focused to detection of these two pollutants for air quality monitoring (AQM) applications. In this regard, there are currently two types of sensing technology available: electrochemical and solid state sensors. The most accurate devices use an electrochemical element [15]. These sensors are gas specific and have high accuracy, but they have a short life-time. Solid state sensors are instead susceptible to temperature and are also cross sensitive to other gases and thus, prone to false alarms. A solution to improve the selectivity of solid state sensors is to use physical or chemical filters placed before the sensing material in order to modify the gas composition reaching the sensing layer. Pijolat and co-workers have firstly tried to use a thin metallic filter made of rhodium above a thin SnO_2_ sensing material for the detection of NO*_x_* with minimal CO interference [16]. Nevertheless, the direct contact between the metallic film and the SnO_2_ can affect the efficiency of such filters, on one hand in relation to a problem of short-circuit for high thickness, and on the other hand under gases with complicated mechanisms as the redox reaction with CO on platinum. To overcome these limitations, more selective sensors which respond to CO and NO_2_ without any interference of the other, are necessary.

A preliminary work, devoted to demonstrate the possibility to detect selectively CO and NO_2_ by means of ZnO-based sensors, is here reported. Even if the morphology of sensing material plays an important role in the gas sensor performance, data in literature on this subject are scarce. Gurlo reviewed, in an recent paper, several case studies related to more important sensing materials such as SnO_2_, ZnO, In_2_O_3_ and WO_3_ [17]. He concluded that a control over crystal morphology, *i.e.*, over size and shape of faces in a crystal, is required for the development of better sensors with increased selectivity and sensitivity. Therefore, the first step toward this morphological control of the gas sensing properties is the design and synthesis of well-defined nanomaterials which are uniform in size, shape and surface structure. For example, Micro-Chemical Systems manufactures dual element sensors for detecting both reducing gases such as CO and HC’s and oxidizing gases such as NO_2_ and O_3_ suitable for air quality monitoring, by designing a sensor sensing layer using proprietary nanostructured metal oxides with special features [18].

In this concern, results obtained with solid state sensors based on ZnO nanomaterials synthesized by sol-gel and electrospinning and having different morphology, are here reported and discussed. The effects of the morphology and annealing temperature on the sensing performances to CO and NO_2_ were studied.

## 2. Results and Discussion

### 2.1. Synthesis and Morphological/Microstructural Characterization

ZnO nanomaterials were obtained by two different synthesis procedures namely the sol-gel and electrospinning methods. Sol-gel technique has been largely used to synthesize metal oxides for sensing applications. Here, ZnO nanopowders have been obtained by a new approach based on slow hydrolysis of the precursor using an esterification reaction in the alcoholic medium, followed by a supercritical drying in ethanol [19]. Thus, powders of ZnO with size in the nanometer range can be easily obtained, which could show interesting properties for gas sensing. The nanopowders obtained were annealed at medium (400 °C) and high (700 °C) temperature.

Electrospinning technique also offers numerous advantages for obtaining metal oxide fibers, such as TiO_2_, MoO_3_, SnO_2_ and WO_3_, for gas sensors. Electrospun metal oxide fibers are reported to be able in detecting low concentration of gaseous substances with high sensitivity [20]. The one-dimensional nanofibers synthesized and annealed at 400 °C provide high surface-to-volume ratio, then much more adsorption sites for gaseous species are expected to be presents on their surface.

**Figure 1:**
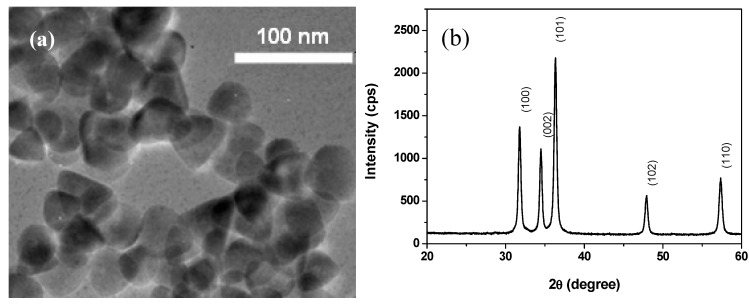
(**a**) TEM images of ZnO NPs; (**b**) X-ray diffraction pattern of these nanoparticles.

To compare the morphological and microstructural characteristics of samples synthesized, Scanning and Transmission Electron Microscopy (SEM, TEM) and X-ray Diffraction (XRD) analysis have been performed. Figure 1a reports a typical TEM image taken from the sample as-prepared by sol-gel in supercritical conditions. Very small ZnO NPs having size in the nanometer range are observed. The crystallites present a prismatic-like shape with a narrow particle size distribution. The majority of ZnO particles have a size of about 25 nm, in good agreement with the mean particle size, *D*, deduced from XRD spectrum (see Figure 1b) and calculated using the Scherrer’s formula:

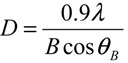
(1)
where *λ* is the X-ray wavelength, *θ_B_* is the maximum of the Bragg diffraction peak (in radians) and *B* is the full width at half maximum (FWHM) of the XRD peak. The spectrum shows only diffraction peaks of ZnO, without any additional diffraction peaks. The indexed peaks are related to the hexagonal wurtzite structure according to JCPDS Data Base [21].

**Figure 2:**
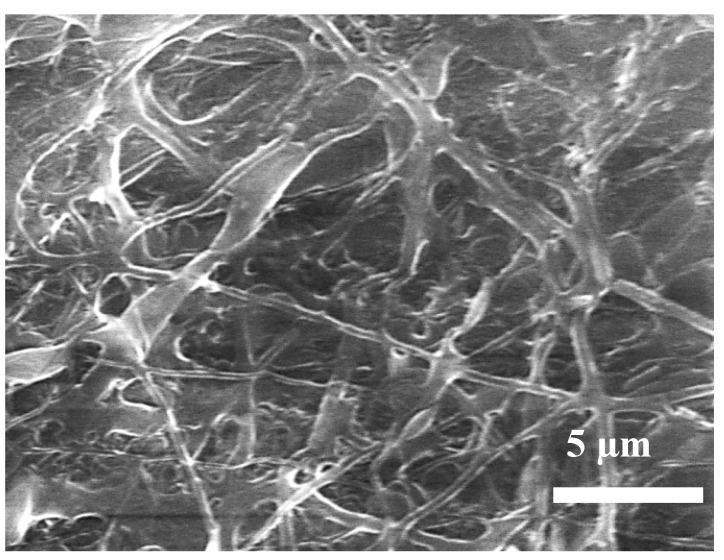
SEM images of ZnO/PVA as-spun nanofibers.

Figure 2 reports a SEM image of electrospun fibers collected directly on the Pt-interdigitated electrode area of alumina sensor support. A network of nanofibers, long several tens of microns can be observed. The electrospun ZnO/PVA fiber mat is composed of irregular fibers having diameter in a large range (100–500 nm), fused among them to form extensive junctions. The distribution of the fibers is fairly random with no distinct preferential alignment.

**Figure 3:**
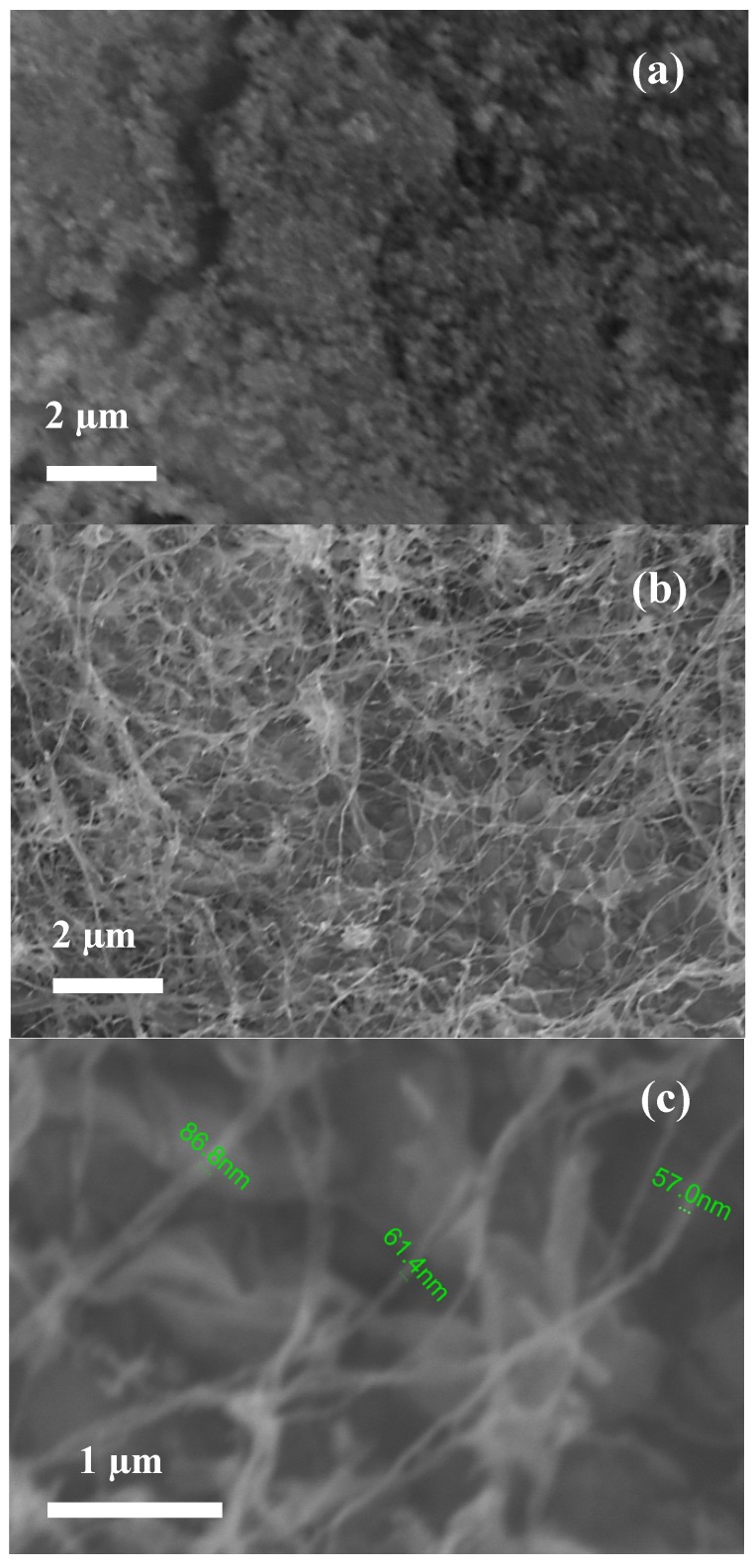
SEM images showing the morphology of samples annealed at 400 °C and deposited on the sensor substrate. (**a**) ZnO NPs; (**b**) ZnO NFs; (**c**) Higher magnification of ZnO NFs.

SEM images showing the morphology of the NPs and NFs after annealing at 400 °C are reported in Figure 3. ZnO NPs particles deposited on the sensor substrate form a porous layer which provides a large number of contacts among the particles (Figure 3a). As a consequence of the elimination of the polymer phase, annealing of the ZnO/PVA fibers (Figure 3b,c), leads to the formation of more homogenous nanofibers with a smaller diameter distribution (50–100 nm).

### 2.2. CO and NO_2_ Sensing Tests

**Figure 4:**
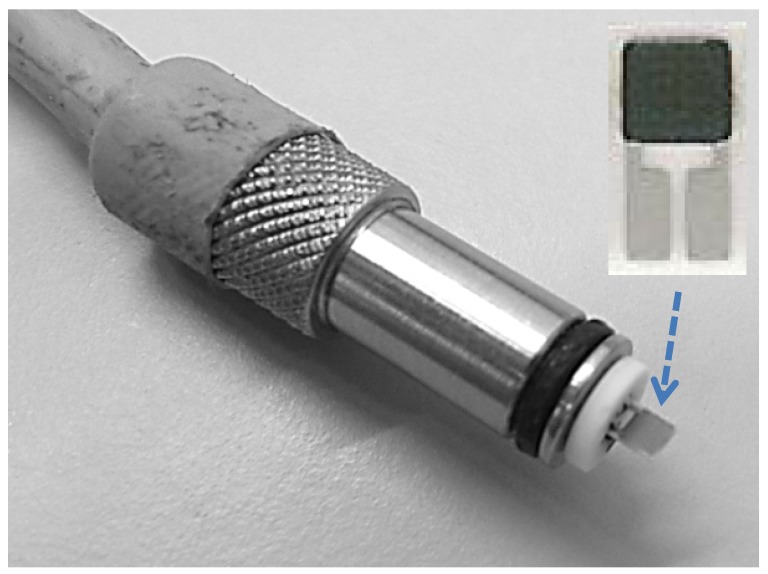
Picture of the home-made probe.

Sensing tests were carried out by means of the home-made sensing probe shown in Figure 4. The sensor head consists of an alumina substrate with Pt interdigitated electrodes. On the back side of alumina substrate, a Pt heater provides to the heating of the sensing element. The active sensing layer was deposited on the Pt interdigitated electrodes area from an aqueous paste of the samples by screen printing. No binder was necessary to enhance the adhesion of the sensing layer on the alumina substrate.

**Figure 5:**
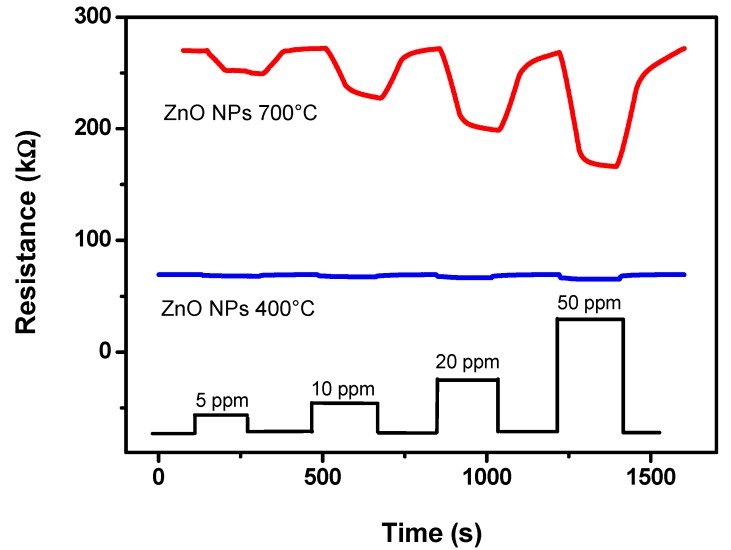
Transient responses to various CO concentrations in air at the operating temperature of 350 °C of the ZnO nanoparticles (NPs) sensor annealed at different temperatures.

In order to simplify the functioning of the dual sensor device, the single sensors would operate at the same temperature. Preliminary work has been then devoted to find the optimal operation temperature. The temperature of 350 °C has been chosen on the basis of the better performance obtained in terms of sensitivity, selectivity and fast response/recovery time. Figure 5 shows the dynamic gas sensing characteristics towards CO of the sensor based on ZnO NPs annealed at 400 °C and 700 °C respectively, at the operating temperature of 350 °C. The resistance decreased upon exposure to 5–50 ppm of CO, in agreement with the generally accepted sensing mechanism on n-type oxide semiconductors, such as SnO_2_, ZnO, and In_2_O_3_. The oxidation reaction at the semiconductor surface between the reducing gas and the negatively charged surface-adsorbed oxygen (O^−^ or O^2−^) is at the basis of this mechanism. This process leads to the production of free electrons as follows:

CO _(g)_ + O^−^_(ads)_ → CO_2_ + e^−^ (2)

The injection of produced electrons from the surface to the bulk of semiconducting layer induces consequently a decrease of the sensor resistance proportional to CO concentration. From the obtained results, it appears that the annealing temperature influence the sensor response towards CO. The ZnO NPs sample annealed at 700 °C shows greater response than the annealed at 400 °C one. XRD analysis showed that the average particle size increase with the annealing temperature, from 25 nm for the as prepared ZnO NPs up to about 66 nm for the sample annealed at 700 °C, leading consequently to a decrease of surface area. Then, it can be deduced that the increase of sensitivity observed for the ZnO NPs sample annealed at the higher temperature tested should be attributed to other factors than the increase of surface area. It is well known that the crystallinity systematically increase with the increase of annealing temperature, as well as the formation of a greater number of oxygen vacancies [22]. These factors then could explain the increased ZnO sensor sensitivity for CO at higher annealing temperature. It is also interesting to note that the detection limit of the sensor based on ZnO NPs annealed at 700 °C, can be estimated to be below 5 ppm of CO in air, a value better than those reported under similar operating conditions for ZnO systems in previous papers [23,24].

**Figure 6:**
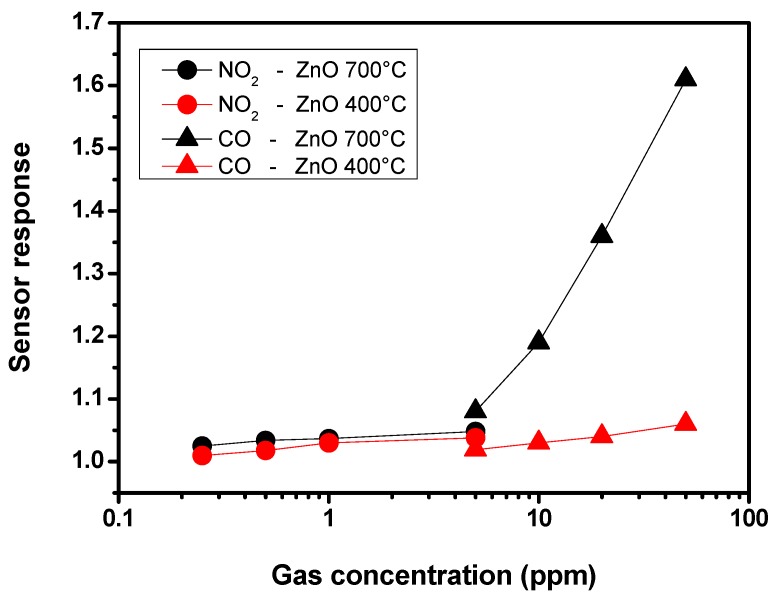
Response of ZnO nanoparticles annealed at 400 °C (red) and 700 °C (black) as a function of CO and NO_2_ concentrations at 350 °C.

These factors have a great impact in favoring the response towards CO, while the response to NO_2_ is unaffected. Indeed, with NO_2_ as target gas at concentrations in the sub-ppm level (≤1 ppm), on ZnO NPs samples annealed at different temperatures, the resistance variations are barely discernible from the baseline. This is clearly observed in the calibration curves, registered at the operating temperature of 350 °C for both CO and NO_2_ gases (Figure 6). Thus, the sensor with NPs annealed at 700 °C results strongly selective towards CO.

**Figure 7:**
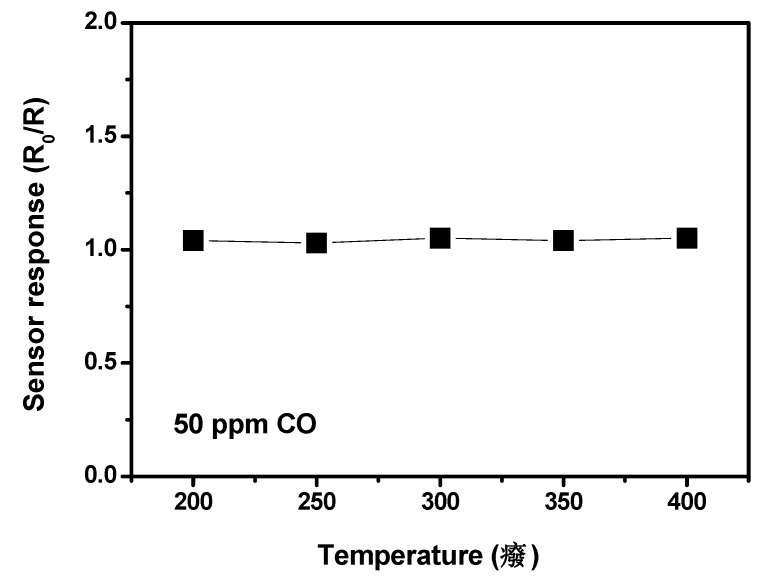
Response of ZnO NFs sensor to 50 ppm of CO as a function of the temperature.

ZnO NFs based sensor behaves instead in a completely opposite way. Specifically, ZnO NFs sensor displayed negligible response to CO in a very large operating temperature range (see Figure 7).

**Figure 8:**
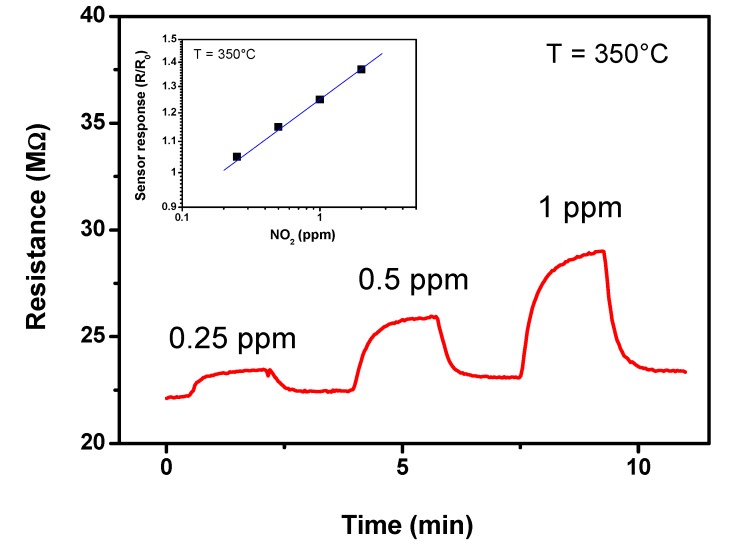
Transient responses of the nanofibers (NFs) based sensor tested to different NO_2_ concentrations in air at the operating temperature of 350 °C. In the inset is shown the calibration curve.

On the other hand, the ZnO NFs sensor has been found sensitive to NO_2_ even at sub-ppm concentrations. Figure 8 shows the dynamic gas sensing characteristics towards NO_2_ of the ZnO NFs-based sensor. The resistance increased upon exposure to NO_2_, suggesting that the detection mechanism can be associated with the following reaction:

NO_2(g)_ + e^−^ → NO + O^−^_(ads)_ (3)

Conduction electrons are then consumed and this leads to an increase of the surface resistance, in agreement with results reported for similar one-dimensional ZnO systems [25]. The linear trend of the sensor response *vs.* the NO_2_ concentration is shown in the inset of Figure 8.

The understanding of the exact mechanism is out of the scope of the present paper. However, some brief considerations are here reported. By the results above reported it appears evident that the annealing temperature and shape of ZnO particles are key factors in determining the sensitivity and selectivity of the sensors investigated. Furthermore, they can also influence the electrical characteristics of the sensing layer. It can be noted that the NPs films are more conductive than the NFs films by two orders of magnitude.

**Figure 9:**
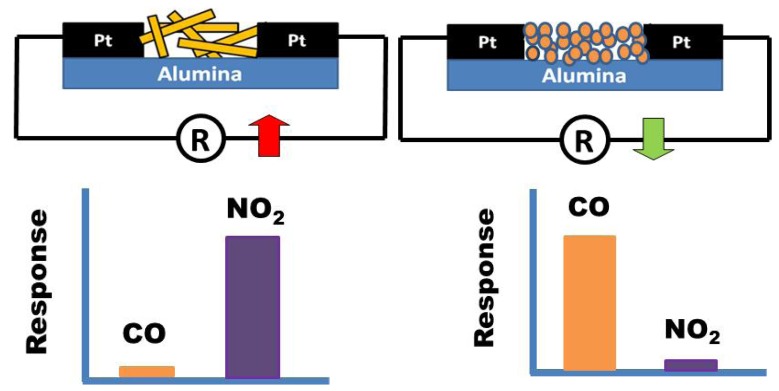
Schematic representation of the distribution of semiconducting particles on the substrate and related sensitivity pattern.

In order to explain this finding, we would consider that the electrical conductivity of polycrystalline metal oxides is mainly governed by the potential barrier formed at the grain boundaries and the electrical conduction occurs along percolation paths via grain boundaries contacts [26]. On theis basis, the difference observed in the electrical resistance baseline value between NPs and NFs can be due to many morphological and microstructural factors which influence, consequently, their electrical properties. Further, the direct deposition of electrospun one-dimensional ZnO NFs, leads to a limited number of contact points established between the nanofibers and the underlying electrode, resulting in a poor electrical contact with the electrode surface. This could give the major contribution to the higher resistance of the ZnO NFs sensor compared to ZnO NPs sensor. A schematization of the sensor device is reported in Figure 9. In the case of NPs, there are many contact points which contributes to the electrical conduction. In contrast, the electrical conduction in NFs is limited by the smaller number of contact points present (see also Figure 3c).

Changes in particle shape can also dramatically alter the reactivity of metal oxides when interacting with gaseous species [12], but no clear correlation has been established with sensing properties comparing the data reported in literature. Nanofibers are generally known to favors the NO_2_ sensitivity. Baratto *et al.*, demonstrated that the fiber structure produces an increase of relative response towards NO_2_ with respect to bulk structure, while they observed no interference from reducing gases such as CO and ethanol [27]. Also Hsueh *et al.*, have shown that ZnO-nanowire based CO sensor presents no response at lower CO concentrations than 500 ppm [28]. This behavior is similar to that reported by us in this work (see the responses to NO_2_ and CO of ZnO NFs reported in Figure 9). In contrast, Lee reported that nanofibers of ZnO show a significant response to CO even at very low concentrations [29]. On these bases, it appears clear that more investigations are necessary in order to better clarify this important issue.

From a practical point of view, the sensor stability for prolonged times is an important prerequisite. The sensor devices have been then tested for prolonged times (around one-two months) and no remarkable degradation of the performances has been noted, indicating the good thermo-mechanical and electrical stability of the sensing layer. Therefore, even if the sensitivity to NO_2_ is lower with respect to similar systems reported in the literature [25], the easy modulation of the sensitivity and selectivity is very promising, making possible the use of the developed ZnO NPs and ZnO NFs single sensor in a dual device designed for the selective monitoring of CO and NO_2_ in ambient air.

## 3. Experimental Details

### 3.1. Samples Preparation

#### 3.1.1. ZnO NFs Preparation

ZnO nanofibers (NFs) were synthesized by electrospinning technique using a suspension of zinc acetate, Zn(CH_3_COO)_2_ 2H_2_O, in poly (vinyl alcohol) (PVA) (Mw = 72.000) as precursors. The main electrospinning process parameters are: the flow rate of 0.1 mL h^−1^ and 8 kV electric potential between the needle and the substrate. By carefully controlling the process parameters of electrospinning, a well reproducible morphology of the ZnO NFs during different depositions can be obtained. The collected nanofibers were annealed in air at 400 °C to obtain pure ZnO NFs.

#### 3.1.2. ZnO NPs Preparation

ZnO nanopowders were prepared by a sol-gel route using zinc acetate as a precursor in methanol. After 15 min under magnetic stirring, the solution was placed in an autoclave and dried in supercritical conditions in presence of ethyl alcohol (*T*_c_ = 243 °C; *P*_c_ = 63.6 bars), according to protocol reported in [19]. In second step, the obtained nanopowders were annealed at 400 and 700 °C.

### 3.2. Characterization

The microstructure of the samples was investigated by XRD (AXS D8 Advance; BRUKER, Billerica, MA, USA) using the Cu *K*_α1_ wavelength of 1.5405 Å. The surface morphology of the films was monitored by means of scanning electron microscopy (SEM) tests carried out with a JEOL 5600LV electron microscope (JEOL, Tokyo, Japan). TEM images were recorded on a Technai G20-Stwin transmission electron microscope (FEI, Eindhoven, The Netherlands) using an accelerating voltage of 200 kV.

### 3.3. Sensing Test

NPs based sensors were made by printing films (1–10 μm thick) of the nano-powders dispersed in water on alumina substrates (6 mm × 3 mm) with Pt interdigitated electrodes and Pt heater located on the backside. NFs based sensors were made by direct spinning of fibers on alumina substrates. The sensors were then introduced in a stainless steel test chamber for the sensing tests. Electrical measurements were carried out in the temperature range from room temperature to 400 °C, under a synthetic dry air total stream of 100 sccm, collecting the sensors resistance data in the four point mode. Gases coming from certified bottles can be further diluted in air at a given concentration by mass flow controllers. The concentration of CO target gas was varied from 5 to 50 ppm, and the concentration of NO_2_ target gas was varied from 0.25 to 2 ppm. A multimeter data acquisition unit Agilent 34970A (Santa Clara, CA, USA) was used for this purpose, while a dual-channel power supplier instrument Agilent E3632A was employed to bias the built-in heater of the sensor to perform measurements at super-ambient temperatures. The gas response, *S*, is defined as *S* = *R*_0_/*R* for CO and *S* = *R*/*R*_0_ for NO_2_, where *R*_0_ is the baseline resistance in dry synthetic air (20% O_2_ in nitrogen) and *R* is the electrical resistance of the sensor at different CO or NO_2_ concentrations, respectively, in dry synthetic air.

## 4. Conclusions

In this work, ZnO nanostructures with different morphologies were synthesized and characterized. Their morphologies and microstructures were determined by SEM, TEM and XRD. ZnO NPs with an average grain size of about 25 nm resulted from the sol-gel synthesis in supercritical conditions. By electrospinning method, ZnO NFs were instead obtained. The response of the synthesized nanomaterials to low concentrations of CO and NO_2_ were measured at working temperatures ranging from 200 to 400 °C. The effects of the annealing temperature and shape on the sensing performances were demonstrated. Sensitive and selective CO sensors based on ZnO NPs annealed at high temperature have been developed, whereas sensitive NO_2_ gas sensors are based on the ZnO NFs synthesized by electrospinning. The developed sensors show promising applications in dual air quality control systems for ambient air and closed ambient conditions, such as automotive cabin, parking garage and tunnels.
